# Lymphocytes and the Dap12 Adaptor Are Key Regulators of Osteoclast Activation Associated with Gonadal Failure

**DOI:** 10.1371/journal.pone.0000585

**Published:** 2007-07-04

**Authors:** Adrienne Anginot, Romain Dacquin, Marlène Mazzorana, Pierre Jurdic

**Affiliations:** Institut de Génomique Fonctionnelle de Lyon, Université de Lyon, Institut Fédératif Biosciences Gerland Lyon Sud, Université Lyon 1, CNRS, INRA, Ecole Normale Supérieure, Lyon, France; University of California, San Francisco, United States of America

## Abstract

Bone resorption by osteoclasts is necessary to maintain bone homeostasis. Osteoclast differentiation from hematopoietic progenitors and their activation depend on M-CSF and RANKL, but also requires co-stimulatory signals acting through receptors associated with DAP12 and FcRγ adaptors. Dap12 mutant mice (KΔ75) are osteopetrotic due to inactive osteoclasts but, surprisingly, these mice are more sensitive than WT mice to bone loss following an ovariectomy. Because estrogen withdrawal is known to disturb bone mass, at least in part, through lymphocyte interaction, we looked at the role of mature lymphocytes on osteoclastogenesis and bone mass in the absence of functional DAP12. Lymphocytes were found to stimulate an early osteoclast differentiation response from Dap12-deficient progenitors *in vitro. In vivo*, Rag1-/- mice lacking mature lymphocytes did not exhibit any bone phenotype, but lost their bone mass after ovariectomy like KΔ75 mice. KΔ75;Rag1-/- double mutant female mice exhibited a more severe osteopetrosis than Dap12-deficient animals but lost their bone mass after ovariectomy, like single mutants. These results suggest that both DAP12 and mature lymphocytes act synergistically to maintain bone mass under physiological conditions, while playing similar but not synergistic co-stimulatory roles in protecting bone loss after gonadal failure. Thus, our data support a role for lymphocytes during osteoclast differentiation and suggest that they may function as accessory cells when regular osteoclast function is compromised.

## Introduction

Bone remodeling and homeostasis result from the balanced activity of two cell types, osteoclasts for bone resorption and osteoblasts for bone formation. In humans, as well as in rodents, bone goes through different phases of development. During the growth phase, bone modeling takes place with a rate of formation higher than that of degradation. Once puberty occurs, the bone mass reaches a peak and subsequently remains constant through a balanced formation and degradation process. Finally, during aging, following gonadal failure and steroid hormone arrest, bone degradation takes over from bone formation leading to osteoporosis [1]. This aging process can be mimicked in animal models such as mice, by gonadectomy, leading to an inhibition of steroid hormone production and decreased bone mass.

In women, the major etiology of osteoporosis is estrogen withdrawal after menopause. Many different studies have been carried out in order to understand the mechanisms of estrogen activity on bone mass, and to generate new therapies to prevent or cure the disease. Despite this work, exactly how estrogens act on bone cells still remains unclear. Most of the estrogen activity on bone cannot be explained by a direct effect on bone cells [2]. Several lines of evidence indicate that disruption of the hormonal status through variations in estrogen levels acts on the hematopoietic compartment and especially on B and T lymphocyte precursors. An elevated estrogen level decreases B lymphopoiesis, whereas a decrease in estrogen levels, following ovariectomy, induces an accumulation of pre-B cells in bone marrow [3]–[5]. Moreover, in the last few years, many reports have emphasized the role played by T lymphocytes through cytokine release, in the control of bone mass under normal physiological conditions or after gonadal failure [6]. T cells have been shown to be one of the key activators of bone resorption via TNFα secretion, leading to osteoporosis in the absence of estrogens [7], [8]. However these data remain controversial as Lee et al. (2006) have recently suggested that T lymphocytes are not implicated in this process [9]. It is currently difficult to have a clear opinion on the precise role of lymphocytes in bone homeostasis, since lymphocytes secrete RANKL, TNFα, IL-1, IL-6, OPG or IFNγ, which have all been shown to have either stimulatory or inhibitory effects on osteoclastogenesis [10].

Mature multinucleated osteoclasts are formed by the fusion of hematopoietic precursors of the myeloid/monocytic lineage. Although, signals driven by Receptor Activator of NF-κB Ligand (RANKL) and Macrophage-Colony-Stimulating Factor (M-CSF) are clearly essential for osteoclastogenesis, regulation by other receptor-mediated signals is less well defined. In addition, recent data have implicated co-stimulatory signals through DAP12 and FcRγ molecular adaptors, which function in concert with many receptors such as TREM2 and OSCAR, respectively [11]. DAP12 (also called KARAP or TYROBP) is a small transmembrane polypeptide comprising a unique cytoplasmic ITAM signaling domain mediating all of its known effector functions. In humans, TREM2 or DAP12 deficiencies are responsible for Nasu-Hakola disease, a rare human disease, characterized at the bone level, by osteoporotic lesions at the extremities of long bones [12], [13]. In contrast, Dap12 complete deletion or Dap12 loss of function (KΔ75) in mice leads to osteopetrosis due to the presence in bones of numerous but inactive multinucleated osteoclasts [14]–[17]. Interestingly, in humans and mice, the bone phenotype appears just after puberty [18], [19] (our unpublished data). Unexpectedly, osteoclast differentiation and function of Nasu-Hakola patient-derived mononuclear myeloid precursors is impaired *in vitro*, similar to the severe block of osteoclast differentiation observed when bone or spleen myeloid precursors from Dap12-deficient mice are cultured in the presence of M-CSF and RANKL. However, co-cultures of Dap12-deficient hematopoietic precursors, together with M-CSF, RANKL and stromal cells or osteoblasts, can restore a weak osteoclastogenesis [14], [16]. *In vivo*, murine osteoclasts differentiate but function poorly whereas, *in vitro*, there is an early block of osteoclastogenesis as no multinucleated osteoclasts can be obtained. Differences between osteoclastogenesis *in vivo* and *in vitro* in humans and in mice, as well as co-culture experiments, suggest that other co-stimulatory signals are present in Dap12-deficient mice to allow osteoclast differentiation. Indeed, Koga et al. (2004) have demonstrated that FcRγ, the other ITAM bearing adaptor protein expressed by osteoclasts, is critical for osteoclast differentiation. Although single FcRγ-/- mice do not present any bone phenotype, Dap12;FcRγ double knock-out mice exhibit a severe osteopetrosis compared to single Dap12-/- mice [14]. Therefore, in the absence of a functional DAP12 protein, FcRγ mediates co-stimulatory signals that can restore a weak osteoclast differentiation. The binding of still unknown ligands to DAP12- and FcRγ- associated receptors leads to the phosphorylation of their ITAM motif, which activates syk kinase and phopholipase Cγ, resulting in the amplification of NFATc1, an osteoclast master gene [14], [15], [20], [21].

Since Dap12 deficiency affects bone resorption after puberty and estrogens regulate the lymphoid cell compartment, we hypothesize that lymphocytes could act as a co-stimulatory signal to stimulate osteoclastogenesis in Dap12 loss of function mice. In this paper, we first show that gonadal failure is a permissive condition that triggers osteoclast resorption in KΔ75 mice. We then address the role played by mature lymphocytes in normal osteoclastogenesis and in bone physiology, analyzing Rag1-/- mice lacking mature B and T lymphocytes, concomitantly with KΔ75 and double mutant Rag1-/-;KΔ75 mice. We show that mature lymphocytes play a similar role to DAP12 in protecting against bone degradation after ovariectomy in female adult mice. Our data support a role for lymphocytes during osteoclastogenesis and suggest that they may function as accessory cells under certain conditions when regular osteoclast function is compromised.

## Results

### Ovariectomy activates bone resorption in Dap12-deficient mice

**Figure 1:**
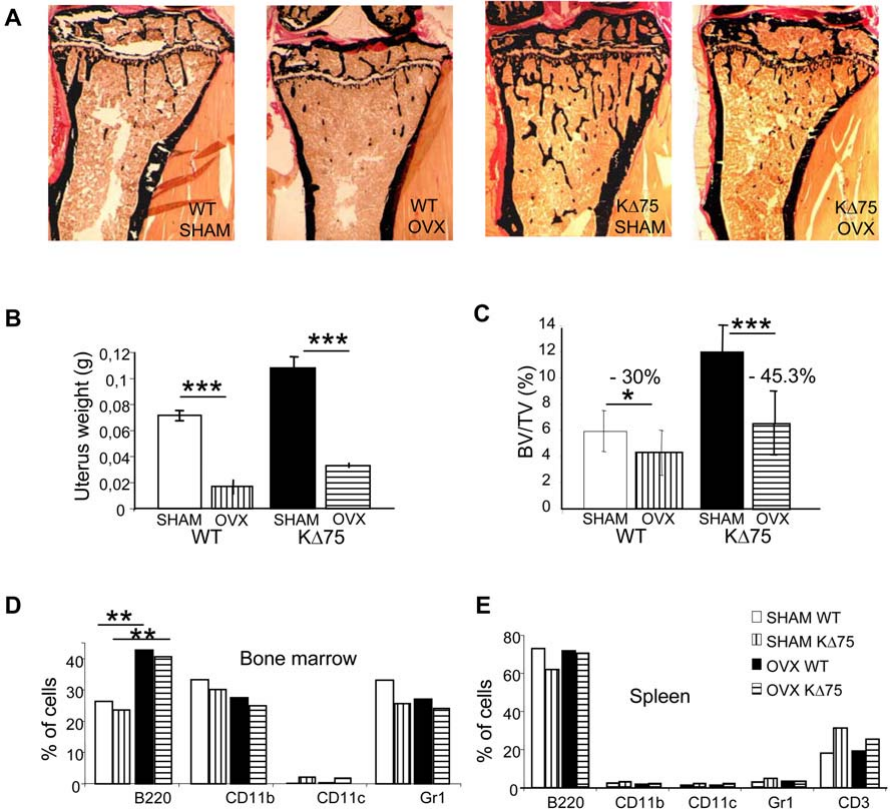
Ovariectomy of KΔ75 mice triggers bone mass reduction. Ovariectomy (ovx) was performed on 8-week-old WT and KΔ75 mice. 4 weeks later, ovariectomized and sham operated animals were sacrificed. (A) Histomorphometric analysis was performed on long bones after Von Kossa/Von Gieson staining. (B) Efficiency of ovariectomy was assessed by weighing uteri. (C) Bone mass was quantified. Results of the quantification show that WT mice lost 30% of bone mass after ovariectomy, whereas KΔ75 mice lost around 45%. (D and E) Hematopoietic populations from bone marrow (D) or spleen (E) were characterized by flow cytometry using the B220 antibody for B lymphocytes; CD11b for monocytes; CD11c for dendritic cells; Gr1 for granulocytes and CD3 for T lymphocytes. (* p<0.01, ** p<0.001, *** p<0.0001)

Since bone mass in Dap12-deficient mice increases due to the inability of osteoclasts to resorb bone after puberty, we wanted to check whether estrogen withdrawal after ovariectomy could reactivate bone resorption and thus restore the bone mass phenotype. For this purpose, 8-week-old KΔ75 and WT female mice were ovariectomized and sacrificed 4 weeks later and their long bones analyzed by histomorphometry (Figure 1A). Ovarian ablation was complete since uteral weight was significantly lower in ovariectomized compared to sham-operated mice (Figure 1B). Bone histomorphometrical analysis showed that the control mice lost 30% of bone mass one month after ovariectomy, but KΔ75 mice lost 45% of their bone mass under the same conditions (Figures 1A and C). This showed that bone resorption, which is impaired in KΔ75 adult mice, can be restored after ovariectomy. Because ovariectomy is known to disturb lymphocyte population homeostasis in the bone marrow we also analyzed the hematopoietic cell distribution in the bone marrow and spleen of KΔ75 mice after ovariectomy. As shown on Figure 1D, only the B220^+^ B lymphocyte number doubled in the bone marrow after ovariectomy in both WT and KΔ75 mice. The numbers of CD11b^+^ monocytes, and Gr1^+^ granulocytes were not modified. In contrast, in spleen, the numbers of B220^+^ B lymphocytes, CD11b^+^ monocytes, CD11c^+^ dendritic cells and Gr1^+^ granulocytes were similar in both KΔ75 and WT mice and were not modified by ovariectomy (Figure 1E). It should be noted that CD11c dendritic cells in bone marrow, as well as CD3^+^ T lymphocytes in spleen had increased in KΔ75 mice but not in response to ovariectomy. This increase in B cell lymphocytes in the bone marrow of ovariectomized female adult mice is in agreement with published data for wild type ovariectomized mice [2], [22]. This prompted us to analyze further the role played by mature lymphocytes on Dap12-deficient osteoclast progenitors.

### Mature lymphocytes enhance osteoclast differentiation of Dap12-deficient progenitors

**Figure 2:**
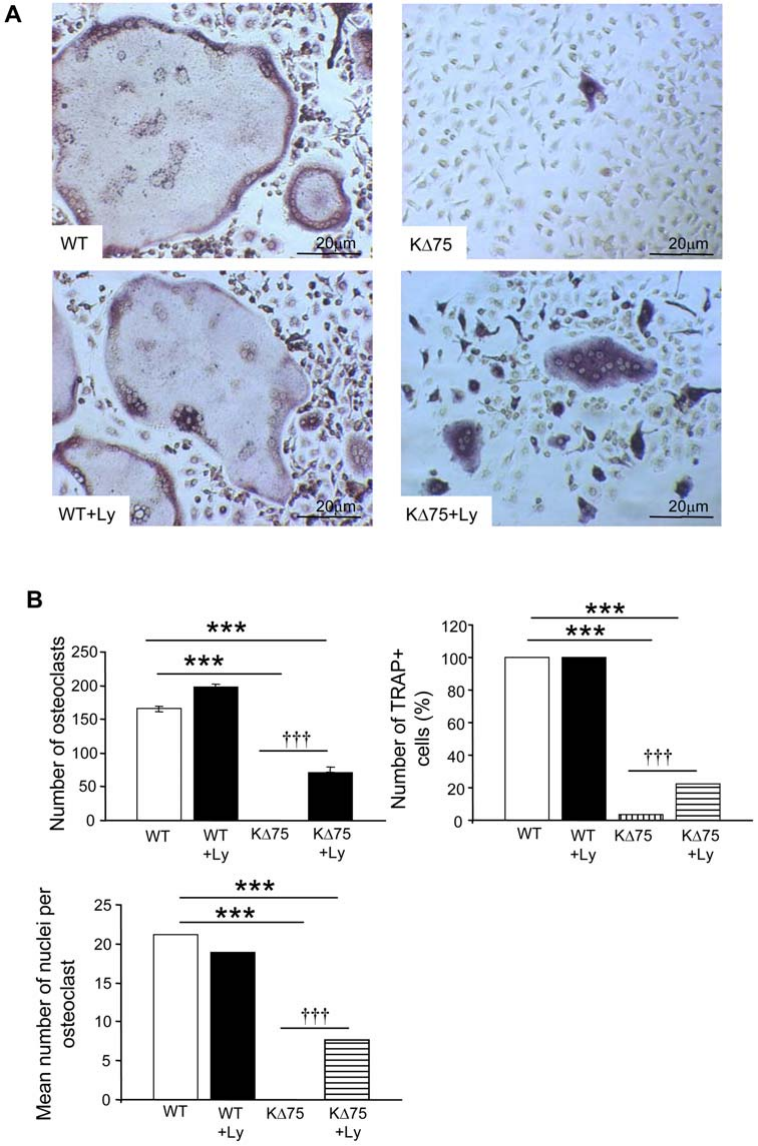
Lymphocytes enhance osteoclastogenesis from KΔ75 but not from WT mouse spleen cells. (A) WT and KΔ75 mice spleen cells were isolated using different lymphocyte separation media to collect osteoclast precursors without (upper panels) or with (lower panels) lymphocytes. Osteoclast differentiation was carried out in the presence of M-CSF and RANKL. Seven days later, cell cultures were fixed and stained for TRAP activity. Lymphocytes increased the formation of TRAP positive osteoclasts from KΔ75 spleen cells (KΔ75+Ly). Pictures of representative fields from several experiments. (B) Quantification under the different culture conditions, of the number of TRAP+ cells per dish; TRAP+ osteoclasts with more than 3 nuclei; average number of nuclei per multinucleated osteoclast. These figures are representative of three different experiments. (* p<0.01, ** p<0.001, *** or ††† p<0.0001)

Osteoclasts are present in Dap12-deficient mouse bones but are poorly functional, whereas *in vitro*, they cannot be differentiated in the presence of MCSF and RANKL, the two osteoclastogenic cytokines. In order to analyze the role of lymphocytes in osteoclast differentiation, we performed *in vitro* osteoclast differentiation from spleen cells from Dap12 loss of function KΔ75 mice versus WT littermates. Leukocytes, in the presence or absence of lymphocytes, were plated in the presence of MCSF and RANKL. In WT mouse cells, osteoclast differentiation occurred independently of the presence of lymphocytes (Figures 2A left panels and 2B). As expected, after 7 days in culture, no osteoclasts differentiated from KΔ75 mouse leukocytes in the absence of lymphocytes. However, when lymphocytes were present in large amounts, we noticed a significant increase in TRAP activity, since 20% of cells in culture were TRAP+. Among these, multinucleated TRAP+ osteoclasts were identified, representing a third of the total number of multinucleated cells formed with WT precursors but containing, on average, 7 nuclei versus 20 in controls (Figures 2A and 2B). This lymphocyte mediated osteoclastogenic effect was not due to RANKL, since addition of soluble RANKL to KΔ75 mouse leukocytes in the absence of lymphocytes did not enhance osteoclast differentiation (data not shown). So, in addition to M-CSF and RANKL, and independently of RANKL concentration, lymphocytes partially restore osteoclast differentiation from Dap12-deficient hematopoietic precursor cells but have no stimulatory role on WT progenitors.

### Mature lymphocytes do not stimulate bone resorption activity of Dap12-deficient osteoclasts

**Figure 3:**
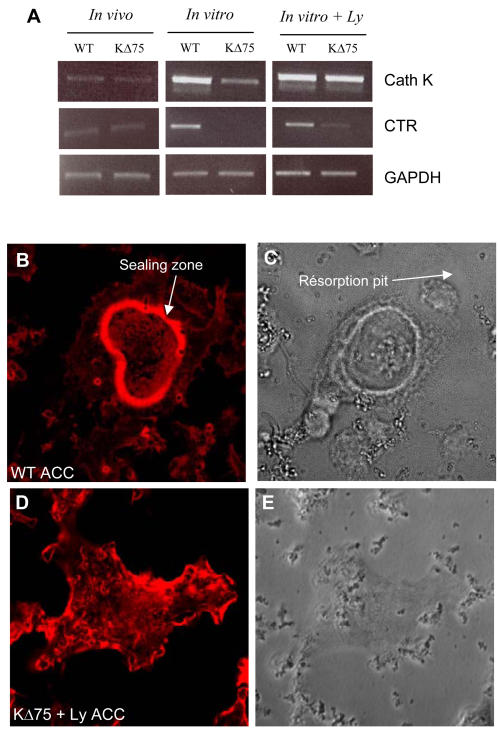
Lymphocytes trigger osteoclast marker expression but not bone resorption from KΔ75 hematopoietic precursors. Expression of cathepsin K (Cath K) and calcitonin (CTR) receptors by RT-PCR. RNAs were extracted from WT and KΔ75 mouse spleen cell cultures (*in vitro*) maintained for 7 days with MCSF and RANKL, with or without (+Ly) lymphocytes. As a control, RNAs from WT and KΔ75 mouse bones (*in vivo*) were also used. Under *in vivo* conditions, both Cath K and CTR were expressed in WT and KΔ75 mouse bones, as well as in *in vitro* cultures from corresponding spleen cell cultures. However, in the absence of lymphocytes in *in vitro* cultures, Cath K is weakly expressed and CTR is absent from KΔ75 mouse cell cultures. (B–E) Functional resorption tests on mineralized ACC matrix. Osteoclasts were differentiated in Petri dishes from WT and KΔ75 mouse spleen cells in the presence of lymphocytes, and then removed before being plated on ACC matrix. After 24h, cell cultures were fixed and stained with phalloidin (B, D) and their resorption ability was evaluated by the formation of pits (C, D). WT osteoclasts on ACC matrix exhibited a typical actin-containing sealing zone associated with resorption (B, C white arrow). In contrast, actin from KΔ75 derived osteoclasts did not form a sealing zone but was mostly arranged in lamellipodia (D) and these did not resorb apatite (E).

Small multinucleated osteoclasts can be differentiated *in vitro* from KΔ75 mouse splenocytes and TRAP activity is increased in the presence of lymphocytes. We then checked whether, in addition to osteoclastogenesis, lymphocytes also stimulate bone resorbing activity of the formed osteoclasts. We evaluated several markers typical of mature functional osteoclasts and compared these activities with those of osteoclasts present in the bones of KΔ75 mice. We first analyzed by RT-PCR the expression of an early differentiation marker (Cathepsin K), and a late differentiation marker (calcitonin receptor, CTR). RNA was extracted from WT and KΔ75 bones without bone marrow (“*in vivo*”) and from WT or KΔ75 mouse spleen cell cultures, enriched or not with lymphocytes (“*in vitro*”). Both Cathepsin K and CTR were expressed at similar levels in bones from WT or KΔ75 mice *in vivo* (Figure 3A). During *in vitro* osteoclast differentiation, cathepsin K was expressed at low levels and CTR was not detected in KΔ75 cells compared to WT cells. However, in lymphocyte enriched cultures, cathepsin K and CTR were expressed by KΔ75 cells, as well as by WT cells (Figure 3A). From these results we can conclude that lymphocytes trigger the expression of differentiation markers of KΔ75 osteoclast precursors. Because the main function of osteoclasts is to resorb bone matrix, we then decided to examine whether or not such differentiated osteoclasts would be able to resorb apatite mineralized matrix like *in vivo* KΔ75 osteoclasts. For this purpose, we analyzed both their ability to form an actin containing sealing zone on calcium apatite and to resorb it. Functional tests for osteoclast activity were carried out on ACC (Apatite Collagen Complex) matrix. Osteoclasts differentiated from WT mouse cells formed a typical sealing zone on the ACC matrices (Figure 3B) and resorbed it, as resorption pits could be observed in the vicinity of osteoclasts (Figure 3C, white arrow). In contrast, osteoclasts differentiated from KΔ75 mouse spleen cells, enriched in lymphocytes and spread on ACC matrix did not form any sealing zone but only actin-containing lamellipodia (Figure 3D). These were not functional since they did not resorb the mineralized matrix (Figure 3E). This absence of resorption was also verified on dentine slices (not shown). So, in the presence of M-CSF and RANKL as well as lymphocytes, spleen cells from KΔ75 mice can differentiate into multinucleated osteoclasts but these are not functional similarly to what is observed *in vivo* inKΔ75 mice. Thus, by adding lymphocytes to *in vitro* cultures we can reproduce the *in vivo* osteoclast phenotype. During osteoclastogenesis, mature lymphocytes may act as accessory cells in the absence of a functional DAP12 protein.

### Absence of mature lymphocytes increases bone mass only in the absence of a functional DAP12 protein

**Figure 4:**
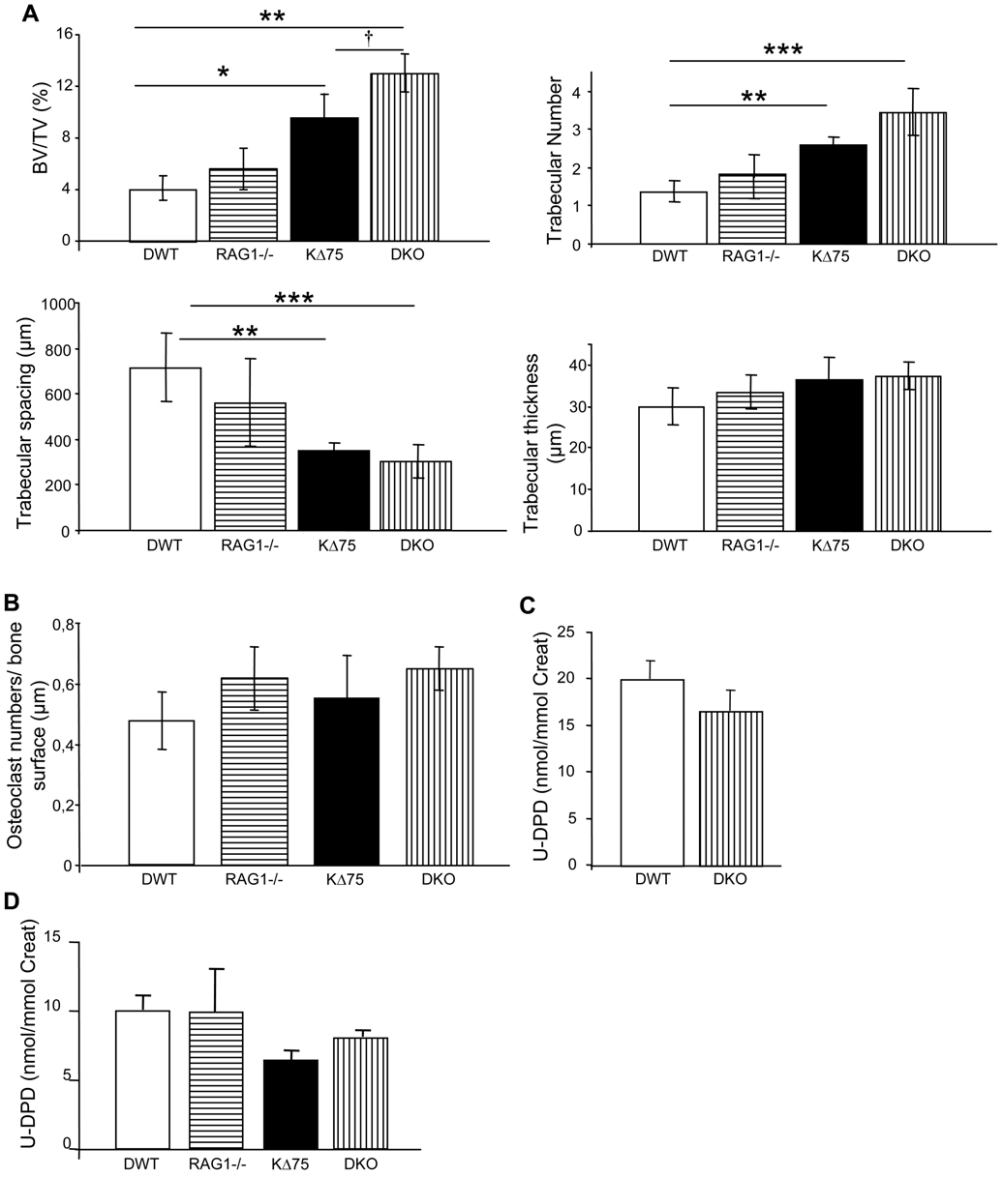
Absence of mature lymphocytes *in vivo* enhances the osteopetrosis phenotype in KΔ75 mice through inhibition of osteoclast function. KΔ75 mice were crossbred with Rag1-/- mice devoid of mature lymphocytes. The bone phenotype of KΔ75;Rag1-/- mice (called DKO), as well as control littermates (DWT, Rag1-/- and KΔ75 mice), was quantified by histomorphometric analysis. (A) Bone mass was assessed after Von Kossa/Von Gieson staining; trabeculae numbers, thickness and space between trabeculae were quantified using specific softwares. KΔ75 mice have a mild but significant osteopetrosis compared with DWT mice. Rag1-/- mice did not exhibit any bone phenotype whereas osteopetrosis in KΔ75 mice increased in the absence of lymphocytes (DKO mice) and was statistically significant, compared to DWT or KΔ75 mice. (B) TRAP^+^ osteoclast numbers per bone surface unit were assessed directly on bone slices. No statistical differences were observed between animals of the four different genotypes. (D and E) Deoxypyridinoline cross-link degradation products were measured in evening urines and expressed as a ratio with creatinine in 3-month-old females (C) and 6-month-old males (D). (*or † p<0.01, ** p<0.001, *** p<0.0001)

As lymphocytes compensate for Dap12 deficiency for *in vitro* osteoclastogenesis, we decided to evaluate the importance of lymphocytes in the control of bone mass. We chose the Rag1-/- mouse model deficient in both mature T and B lymphocytes [23]. We generated Rag1-/- or KΔ75 as well as Rag1-/-;KΔ75 (DKO) double mutant mice with identical genetic backgrounds. The bone parameters (BV/TV) of 12-week-old female mice were analyzed first with a micro scanner (Data S1) and also, for confirmation, by histomorphometry on calcified bones (Figure 4A and B). We first observed that Rag1-/- mice did not present any particular bone phenotype in comparison to WT animals of a similar genotype. This genetic approach allowed us to confirm once again that KΔ75 mice had an increased bone mass (21%) compared with WT or Rag1-/- mice. More importantly, we observed that DKO mice had an even higher increased bone mass to compare with KΔ75 (30%) and control or Rag1-/- mice (60%) (Figure 4A, Table 1). In comparison with other mice, trabeculae thickness was unchanged in the DKO animals. In addition, the number of trabaculae had increased, whereas the space between them was reduced. This phenotype is mostly due to impairment of osteoclast function, since the number of osteoclasts counted on bone surfaces was similar for all four backgrounds (Figure 4B), while collagen type I degradation products present in the urine of 12-week-old DKO female mice had decreased by 17% compared with WT females (Figure 4C). This was further confirmed in 6-month-old male mice in which collagen type I degradation products had decreased in both KΔ75 and DKO mice, compared with WT and Rag1-/- mice (Figure 4D). These data demonstrate that the absence of mature B and T lymphocytes does not affect bone mass in mice with a normal genetic background but acts synergistically with a Dap12 deficiency to increase bone mass by inhibiting osteoclast activity (Table 1).

**Table 1:** Summary of bone mass phenotype in normal or ovariectomized 3-month-old female mice according to genotype

Mice genotype	DWT	KΔ75	Rag1 -/-	DKO
**Bone mass**	**Normal**	**Mild osteopetrosis**	**Normal**	**Severe osteopetrosis**
**Bone mass after ovariectomy**	**Reduced (30%)**	**Highly reduced (50%)**	**Highly reduced (50%)**	**Highly reduced (50%)**

### Mature lymphocytes are not implicated in bone loss after ovariectomy

**Figure 5:**
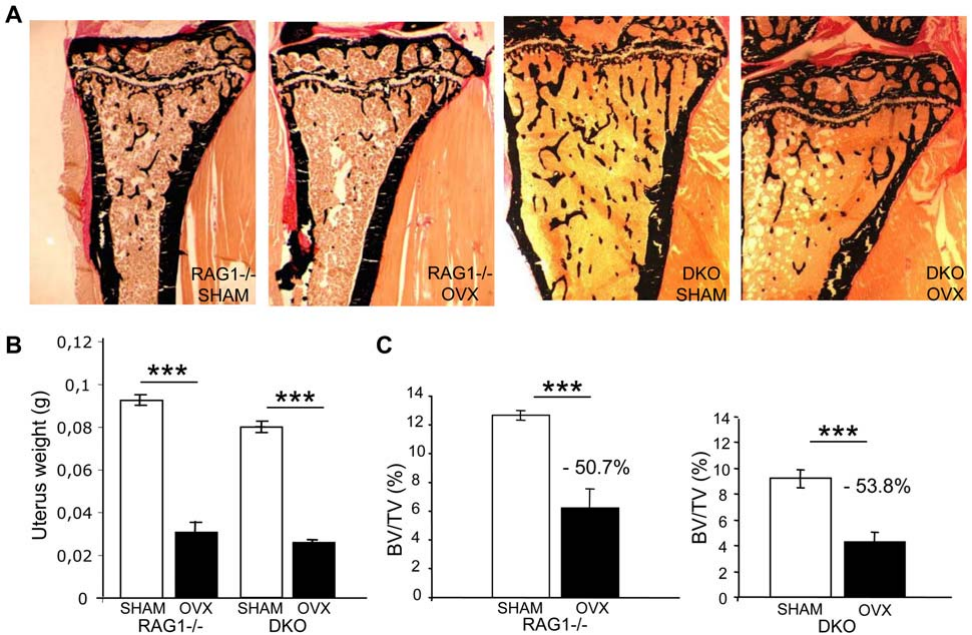
Mature lymphocytes and DAP12 protect against gonadal failure induced bone loss. Ovariectomies were performed on 8-week-old Rag1-/- and DKO mice. Four weeks later, both ovariectomized and sham operated animals were sacrificed. (A) Bone mass was assessed by histomorphometric analysis after Von Kossa/Von Gieson staining. (B) The efficiency of ovariectomy was evaluated by weighing the uteri. (C) Results of the quantification show that Rag1-/- mice, as well as KΔ75;Rag1-/- mice, lost more than 50% of their bone mass after ovariectomy. (*** or ††† p<0.0001)

As DAP12 activity protects against bone loss after ovariectomy and lymphocytes seem to play a similar accessory role, we wanted to know whether Dap12 deletion would act independently or synergistically with mature lymphocyte depletion. Eight-week-old Rag1-/- and DKO female mice were ovariectomized or sham operated, and one month later were sacrificed for bone histomorphometry analysis (Figure 5A). The uteri of sacrificed sham operated and ovariectomized animals were weighed to ensure that the ovariectomy had been successful (Figure 5B). BV/TV evaluated by microscanner imaging (not shown) and histomorphometry analysis showed that Rag1-/- mice had lost more than 50% of their bone mass (Figure 5C) compared with the 30% lost by WT female mice (Figure 1C), suggesting that mature lymphocytes play an important role in protecting against bone loss after gonadectomy (Table 1). Double mutant mice deficient in both Dap12 and mature lymphocytes also lost 50% of their bone mass (Figure 5C), compared with 45% bone loss after ovariectomy of KΔ75 mice (Figure 1C). In summary, it is possible to compare levels of bone loss after ovariectomy in the absence of mature lymphocytes and in the presence or absence of functional DAP12 protein (Data S2). Strikingly, although WT female mice lost 30% of bone mass after ovariectomy, the bone loss was 45 to 50% for mice lacking lymphocytes in the presence or absence of a functional Dap12 gene. In addition, the protective effects of DAP12 and lymphocytes on bone mass loss after ovariectomy were not cumulative (Table 1).

## Discussion

DAP12 is a molecular adaptor for transmembrane receptors of unknown ligands, which signals through an ITAM domain. It is required for normal bone homeostasis because mice with impaired DAP12 function, either by targeted inactivation of the ITAM domain or through its complete deletion, develop osteopetrosis after puberty due to the inability of osteoclasts to resorb bone. In this study, using Dap12 ITAM-deficient mice (KΔ75), we expanded on these previous data by showing unexpectedly that bone resorption in mutant adult female mice is restored after ovariectomy. KΔ75 adult female mice were much more sensitive to ovariectomy than WT littermates. Looking for potential cofactors that could act together with DAP12, to maintain bone homeostasis during adult life, we turned our interest toward lymphocytes. Adult Rag1-/- mice, which do not produce any mature B and T lymphocytes, do not present any bone phenotype but suffer a massive decrease in bone mass after ovariectomy. However, double mutant mice with an inactivated DAP12 protein in the absence of mature lymphocytes had increased osteopetrosis compared with KΔ75 mice and their bone mass was decreased to the same extent as KΔ75 animals after ovariectomy (Table 1).

Increasing evidence suggests that immune and skeletal systems share a number of regulatory molecules, but the role played by lymphocytes in bone homeostasis is still controversial [6], [24]. It has been proposed that T lymphocytes are important players in postmenopausal osteoporosis [25] but opposing data have also been published [9]. T lymphocytes could act directly or indirectly on bone cells and mostly on osteoclasts through the secretion of various cytokines. However, even this is difficult to reconcile since T lymphocytes have been reported to secrete cytokines with osteoclastogenic activities such as RANKL, TNFα, IL1, IL6 or antiosteoclastogenic factors such as OPG. The effects of T cells on osteoclastogenesis should depend on the balance between the positive and negative factors that they express. For example, T cell-produced IFNγ which has been reported to inhibit osteoclastogenesis directly but to stimulate it indirectly through antigen-driven T cell activation [26], [27]. On the other hand, the role played by B lymphocytes is mostly recognized through the observation that B lymphopoiesis is stimulated during estrogen deficiency [22], [28]. We reconfirmed this although only in the bone marrow of ovariectomized mice and not in spleen. It is therefore extremely difficult to reconcile all these contradictory data, which could also depend on the genetic background of animals studied. It is also possible to propose that the co-stimulatory pathways involved in osteoclastogenesis could play different, or even opposite roles in specific anatomical bone microenvironments as Wu et al. have shown for ITAM-bearing coadaptors [Wu et al., 2007, PLOSone]. In this work we show that in the specific context of Rag1-deficient mice, mature B and T lymphocytes do not play any role in normal adult bone homeostasis but overall bone mass is drastically reduced after ovariectomy. We cannot rule out the possibility that T and B lymphocytes play opposite roles, which are then nullified in the absence of the two populations. To decipher the role played by lymphocytes in bone homeostasis the specific role of lymphocyte subpopulations will have to be addressed. Indeed, a recent report by Sato et al. has shown the specific importance of the T helper 17 (Th17) subpopulation through secretion of IL17 in bone destruction in rheumatoid arthritis [29].

The protective effect of DAP12 on ovariectomy-induced bone loss has been emphasized since we show here that bone resorption activity can be restored after ovariectomy of Dap12 loss of function mutant mice. Similar results have been obtained with female mice in which Dap12 has been completely deleted [Wu et al., 2007, PLOSone]. In the physiopathological context of gonadal failure, DAP12 impairment and lymphocyte depletion do not act synergistically since bone mass reduction after ovariectomy is the same when either one or both of these factors are disturbed (Data S2). However, B and T lymphocytes can partially compensate for the lack of DAP12 *in vitro* and *in vivo*. Indeed, from our *in vitro* studies we have shown that lymphocytes enhance Dap12-deficient osteoclast precursor differentiation but do not activate their bone resorbing activity like osteoblasts or stromal cells [15], [16], [19]. This supports the observation that *in vivo*, the inhibition of osteoclast activity in KΔ75 mice is more drastic in the absence of lymphocytes, leading to a more severe osteopetrosis, reinforcing the hypothesis that lymphocytes are somehow implicated in the control of bone mass reduction in relation to Dap12 deficiency.

From our results we can propose that lymphocytes play a similar role to the FcRγ co-stimulatory pathway on osteoclastogenesis and bone mass homeostasis [14], [15], [30]. Indeed, the absence of mature lymphocytes *in vivo* mimics FcRγ deficiency as far as bone mass homeostasis is concerned, according to data reported by Wu et al. (accompanying article). It is then possible to conclude that Dap12 and mature lymphocytes play similar roles in protecting bone mass in the specific context of gonadal failure.

## Materials and Methods

### Mice

KΔ75 loss-of-function mice were derived from eight backcrosses with C57BL/6 mice [31]. KΔ75 mice and Rag1-/- (C57Bl/10) (Charles River Laboratories) mice were crossbred to obtain double heterozygotes. All mice used came from the crossbreeding of the double heterozygote mice. They were bred and kept under pathogen free conditions with sterilized food, water and bedding in the Plateau de Biologie Experimentale de la Souris (IFR128 Lyon Biosciences, Lyon, France). To induce sex hormone deficiency, 8-week-old female mice underwent transabdominal ovariectomies (OVX), whereas the controls consisted of sham-operated mice (SHAM). Surgery was performed under general anesthesia using a Xylasine (12 mg/kg, Sigma)/Ketamine hydrochloride (0.1 mg/kg, Centravet) mixture, following procedures in agreement with the ethical rules set by the IFR 128 animal facility committee.

### Cell cultures

Leukocytes were isolated from 8-week-old mouse spleens. Spleens were dissociated in complete medium (αMEM (Life technologies), 10% serum (Biowest), 2 mM L-Glutamine, 100U/mL Penicillin, 100 µg/ml streptomycin (Invitrogen)), then filtered through a 100 µm cell strainer (Falcon). Two lymphocyte separation media with different densities were used to isolate leukocytes: Eurobio (density 1.077 g/cm^3^) and Lympholyte-M (density 1.0875 g/cm^3^; Cederlane) (The high density medium allowed lymphocytes to be excluded from nucleated spleen cells such as monocytes, NK cells or dendritic cells. Leukocytes isolated from WT and KΔ75 mice, plated at 2500 cells/mm^2^, or from Rag1-/- and double mutant mouse cells plated at 250 cells/mm^2^ were cultured in complete αMEM in the presence of M-CSF and RANKL. Osteoclast differentiation was evaluated by Tartrate Resistant Acid Phosphatase staining, using the Leucocyte Acid Phosphatase kit (Sigma). Recombinant murine M-CSF and human RANKL were produced in our laboratory using insect cells baculovirus system and Pichia pastoris recombinant yeast cells, respectively [32].

Apatite Collagen Complex (ACC) coverslips to test osteoclast resorption ability are composed of glass coverslips coated with type I collagen and mineralized by calcium apatite crystals, which can be resorbed by mature osteoclasts. They were prepared according to a method described previously [33], [34].

### Immunofluorescence

Osteoclasts differentiated from spleen leucocytes in the presence of M-CSF and RANKL, were detached with PBS EDTA (0.25 mM) and plated on ACC slices overnight at 37°C. Cells were then fixed in 4% paraformaldehyde (pH 7.2) for 15min at 4°C and rinsed with PBS. They were then labeled with phalloidine-Alexa 546 and observed with a confocal microscope (model LSM510; Carl Zeiss MicroImaging, Inc.). Resorption pits were directly observed by phase contrast microscopy.

### Reverse Transcriptase and Polymerase Chain Reaction

mRNA was extracted with RNAnow reagent (Biogentex) either from *in vitro* cell cultures, or directly from bones devoid of marrow, crushed in liquid nitrogen. Aliquots (1 µg) were reverse transcribed using the MuLV-RT kit (Promega). Polymerase chain reactions were performed with specific primers using the Taq polymerase kit (Eurobio). Oligonucleotides were designed as follows:

-GAPDH 5′-CAAAGTGGAGATTGTTGCCAT-3′

3′-CACCACCTTCTTGATGTCATC-5′

-Cathepsin K 5′-TTAATTTGGGAGAAAAACCT-3′

3′-AGCCGCCTCCACAGCCATAAT-5′

-Calcitonin receptor 5′-TGGTGGAGGTTGTGCCCAATGGAGA-3′

3′-CTCGTGGGTTTGCCTCATCTTGGTC-5′

### Bone Parameters and Histomorphometric Analysis

Bones were fixed in PBS plus 4% paraformaldehyde over night at 4°C and then stored in 70% ethanol. Femurs were scanned by high resolution micro-CT (µCT-20, Scanco Medical, Bassersdorf, Switzerland) with a cubic voxel size of 9 µm and the 3D structural parameters calculated. For histomorphometric analysis, calcified bones were fixed and embedded in methylmethacrylate according to standard protocols [35]. Seven µm thick methyl methacrylate sections were used for Von Gieson/Von Kossa staining as well as for TRAP/hematoxylin stainings. Bone cellular and macroscopic measurements were made using a microscope (model DMLB; Leica), a 3CCD color video DXC-390 camera (Sony) and the OsteoMeasure Analysis System (Osteometrics). Bone-related degradation products from type 1 collagen, deoxypyridinoline cross-links and creatinine were measured in evening urine using the Pyrilinks-D immunoassay and creatine kit (Quidel corporation, San Diego, CA), according to the manufacturer's protocols.

Animal groups were composed of a minimum of 6 to 8 mice. Statistical differences between groups were assessed using the Students *t* test.

## Supporting Information

Data S1 The bone phenotype of DKO, Rag1-/- and K Δ 75 mice and control littermates DWT was imaged (A) and quantified (B) by microscanner in order to confirm the histomorphometric analysis (Figure 4). K Δ 75 mice exhibited mild but significant osteopetrosis compared with DWT mice. Rag1-/- mice did not exhibit any significant bone phenotype whereas the osteopetrosis of K Δ 75 mice had increased in the absence of lymphocytes (DKO mice). (* p<0.01, ** p<0.001)
(2.43 MB TIF)

Data S2 Percentage of bone mass loss after ovariectomy in 3-month-old female mice of identical genetic background.
(0.22 MB TIF)
