# Supplementary figures and images for: Lymphocytes and the Dap12 Adaptor Are Key Regulators of Osteoclast Activation Associated with Gonadal Failure
Source: PLoS One. 2007 Jul 4;2(7):e585. doi: 10.1371/journal.pone.0000585 (PMC1899087; doi:10.1371/journal.pone.0000585)

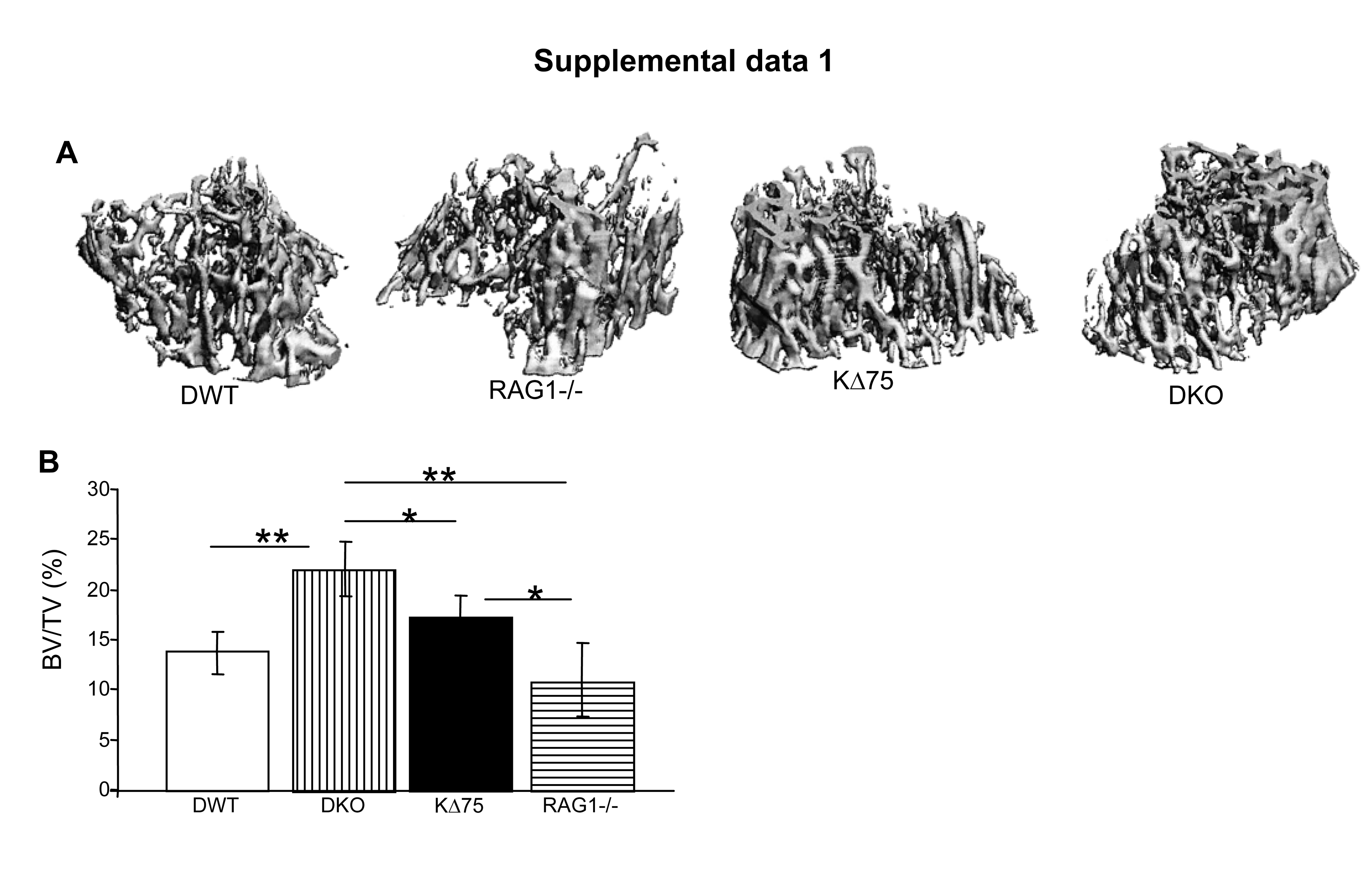

Supplement: Data S1 — The bone phenotype of DKO, Rag1-/- and K Δ 75 mice and control littermates DWT was imaged (A) and quantified (B) by microscanner in order to confirm the histomorphometric analysis (Figure 4). K Δ 75 mice exhibited mild but significant osteopetrosis compared with DWT mice. Rag1-/- mice did not exhibit any significant bone phenotype whereas the osteopetrosis of K Δ 75 mice had increased in the absence of lymphocytes (DKO mice). (* p<0.01, ** p<0.001) (2.43 MB TIF) [file pone.0000585.s001.tif]

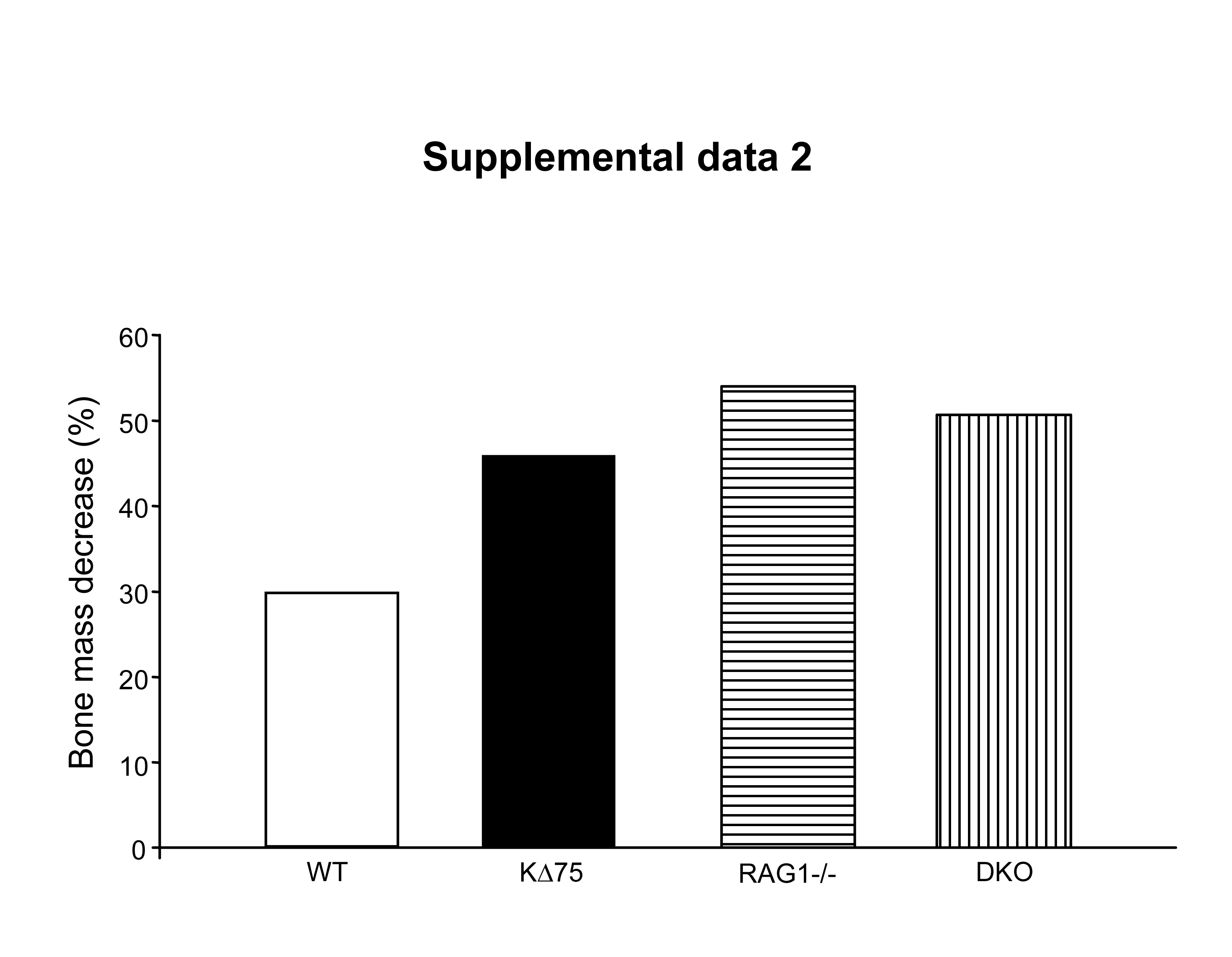

Supplement: Data S2 — Percentage of bone mass loss after ovariectomy in 3-month-old female mice of identical genetic background. (0.22 MB TIF) [file pone.0000585.s002.tif]
